# Diagnosis of Pyomyositis in a Pediatric Patient with Point-of-Care Ultrasound

**DOI:** 10.5811/westjem.2016.5.30331

**Published:** 2016-06-22

**Authors:** Eugene Park, Mikaela Chilstrom

**Affiliations:** LAC + USC Medical Center, Department of Emergency Medicine, Los Angeles, California

## CASE DESCRIPTION

A three-year-old girl presented to the emergency department (ED) for five days of pain and decreased mobility of the left shoulder. She had been evaluated in the ED five days prior for shoulder pain after a minor slip and fall with negative clavicle radiographs, and was discharged home with supportive care. Since the initial visit, her shoulder pain increased and she would not use her arm. Physical examination demonstrated subtle swelling of the left anterior shoulder without erythema, warmth, or fluctuance. Her exam yielded mild tenderness to palpation and markedly decreased range of motion secondary to pain. Point-of-care shoulder ultrasound revealed an enlarged deltoid muscle with a heterogeneous fluid collection within the muscle, but no joint effusion (Video).

## DIAGNOSIS

### Pyomyositis of deltoid and pectoralis major muscles

Based on the ultrasound results, magnetic resonance imaging of the shoulder was ordered, which showed a multiloculated fluid collection within the anterior head of the deltoid and distal pectoralis major muscles consistent with pyomyositis and abscess (Figure). The patient was admitted on parenteral antibiotics; cultures from an incision and drainage grew oxacillin-resistant Staphylococcus aureus. By post-operative day 4, she had complete resolution of her symptoms.

Pyomyositis is an infection of skeletal muscle commonly associated with abscess formation. It is a rare disease in the United States, but is common in tropical areas.^1^ The pathogenesis is unknown but is speculated to develop secondary to hematogenous spread from transient bacteremia, likely in the setting of minor skeletal muscle injury.^2^ Common bacteria implicated are skin flora; antibiotic coverage for methicillin-resistant *S. aureus* and streptococci is recommended. However, in immunocompromised individuals, broad-spectrum coverage is warranted. When combined with surgical incision and drainage, complete resolution can be expected in the majority of cases.

## Supplementary Information

Video Sagittal ultrasound of the left shoulder demonstrates a heterogenous fluid collection within the deltoid muscle.

## Figures and Tables

**Figure:**
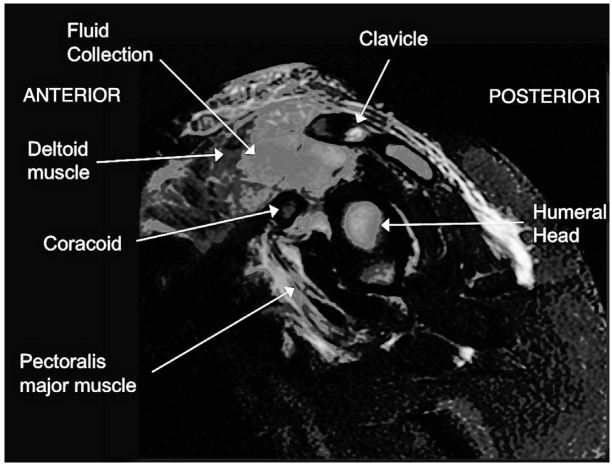
Sagittal magnetic resonance imaging of the left shoulder shows a multiloculated fluid collection within the anterior head of the deltoid and distal pectoralis major muscles.
